# Supplementary material for: A neuron-glia circuit anticipates hypoxia to regulate organismal oxygen use
Source: bioRxiv. 2026 Apr 14:2026.04.10.717666. Preprint. [Version 1] doi: 10.64898/2026.04.10.717666 (PMC13104814; doi:10.64898/2026.04.10.717666)
Supplement: Supplementary file 1 [file NIHPP2026.04.10.717666v1-supplement-2.pdf]

bioRxiv preprint doi: https://doi.org/10.64898/2026.04.10.717666; this version posted April 14, 2026. The copyright holder for this preprint (which was not certified by peer review) is the author/funder, who has granted bioRxiv a license to display the preprint in perpetuity. It is made available under aCC-BY 4.0 International license.

Supplementary information

Supplementary Methods

Cellular model for NE-MO dynamics

We used a generalized leaky integrate-and-fire model, GLIF (Teeter et al, 2018), to simulate the NE-MO cell in the whole-cell patch-clamp recordings (Fig. 3e; Extended Data Fig. 5h,i). The model was made up of two parts: a linear dynamics of voltage change to input and a dynamics of the firing threshold:

$$V'(t) = -\frac{1}{\tau}(V(t) - E_L) + gI$$

$$\Theta'(t) = a(V(t) - E_L) - b\Theta(t)$$

E_L is the resting membrane potential, $\tau = 20$ ms, $b = 0.009$ /ms are time constants of membrane potential and voltage dependence of the threshold, respectively, and $a = 0.0001$ /ms couples the membrane potential to the threshold. I is the external current and g=1 µS is the impedance.

If $V(t) > \Theta(t) + \Theta_\infty$, a spike is generated, and then $V(t)$ was reset as follow:

$$V(t_+) = E_L + f_v(V(t_-) - E_L) - \delta V$$

$$\Theta(t_+) = \Theta(t_-) + \delta\Theta$$

where $f_v = 0.6$, and $\delta V = -5$ mV, are the slope and intercept of the linear relationship of the voltage before and after a spike, respectively; $\Theta_\infty = -39$ mV is the baseline firing threshold; the transient increase of the firing threshold after a spike event $\delta\Theta = 4$ mV reflects the repolarization or after-hyperpolarization which inhibits spike generation after a spike.

In the simulation, we used $E_L = -61$ mV for normoxia and $E_L = -57$ mV for hypoxia, respectively. A linear increase of E_L from -61 mV to -57 mV presents the dynamics of oxygen level transiting from normoxia to hypoxia. Tonic spikes were measured when external input $I = 0$ pA, while phasic spikes were measured when a transient pulse $I = 9$ pA was provided for 300 ms every 3 seconds.

Cellular model fits to fluorescence dynamics

bioRxiv preprint doi: https://doi.org/10.64898/2026.04.10.717666; this version posted April 14, 2026. The copyright holder for this preprint (which was not certified by peer review) is the author/funder, who has granted bioRxiv a license to display the preprint in perpetuity. It is made available under aCC-BY 4.0 International license.

We fitted our cellular model to fluorescence dynamics (Extended Data Fig. 5j,k), $\Delta F/F(t)$, every 1 minute:

$$\Delta F/F(t) = f_{pO_2} g(s(t))$$

$$g(s(t)) = \int_{t-\Delta T}^{t} \sqrt{s(x)} exp((x-t)/\tau_s) dx$$

f_{pO_2} is the transform scalar (which depends on O_2 level) from swim to fluorescence; $g(\cdot)$ is an exponential filter function of the square root of swim power in a time window ΔT before the fluorescence. The performance of the fitting was evaluated using variance explained as

$$EV = 1 - < \frac{(\Delta F/F(t) - f_{pO_2} g(s(t)))^2}{(\Delta F/F(t) - < \Delta F/F(t) >_t)^2} >$$

where $<\cdot>$ is the arithmetic average.

Brain-body control system model

We include an alternative model of how external oxygen (O_2) replenishes blood O_2, which in turn supplies muscle O_2 through circulation which leads to qualitatively similar solutions as presented in the main text. Let X, M, D denote blood O_2, muscle O_2, and instantaneous oxygen demand. Their stochastic dynamics are:

$$\dot{X} = \alpha (X^e - X) - \beta (X - M) - m_r$$

$$\dot{M} = \beta (X - M) - D$$

$$\dot{D} = -\gamma D + j_s s$$

where $s dt \sim Poiss(u)$ represents the probability of swimming, given a control rate u. Here, α and β describe oxygen exchange between environment–blood and blood–muscle, γ is the decay of oxygen demand, m_r is metabolic rate, j_s the swim intensity, and X^e the external O_2 level.

The system aims to keep blood O_2 near an optimal setpoint X* while minimizing deviations from a preferred swimming rate u*:

$$l(X, u) = (X - X^*)^2 + \lambda (u - u^*)^2, \qquad \lambda > 0$$

Approximating s by its mean rate u and defining centered variables $\hat{X} = X - X^*$, $\hat{u} = u - u^*$, the system becomes affine-linear. The optimal controller is of LQR form (Anderson & Moore, 2007):

$$u_t = u^* - k_1(X - X^*) - k_2 M - k_3 D$$

The constants k_1, k_2, k_3 depend on the parameters α, β, γ. In our model simulations, we found $k_3 \approx 0$ consistently, and we will therefore exclude it and simplify the controller as

bioRxiv preprint doi: https://doi.org/10.64898/2026.04.10.717666; this version posted April 14, 2026. The copyright holder for this preprint (which was not certified by peer review) is the author/funder, who has granted bioRxiv a license to display the preprint in perpetuity. It is made available under aCC-BY 4.0 International license.

$$u_t = u^* - k_1(X - X^*) - k_2 M$$

The controller directly senses blood O_2 (X) and demand (D) but not muscle O_2 (M), making the system only partially observable. For partially observable systems, the optimal strategy obeys the separation principle, where the optimal controller consists of an optimal state estimation, such as the Kalman filter, and an optimal deterministic control. Therefore, we postulate that the missing variable M can be approximated by a leaky integration of D:

$$\dot{M}_k \approx \beta M_k + \beta X - D.$$

M_k is the estimated muscle oxygen. Finally, to prevent unbounded control, we introduce a sigmoid nonlinearity approximately linear near equilibrium:

$$f(X, M_k) = (1 + \exp[k_1(X - X^*) + k_2 M_k])^{-1},$$

$$u_t = f(X, M_k)\, u^*$$

This formulation captures a simple closed-loop strategy for oxygen homeostasis under stochastic behavioral drive with signals X and M.

References:

Anderson, B. D., & Moore, J. B. (2007). Optimal control: linear quadratic methods. Courier Corporation.

Teeter, C., Iyer, R., Menon, V., Gouwens, N., Feng, D., Berg, J., et al. (2018). Generalized leaky integrate-and-fire models classify multiple neuron types. Nature communications, 9(1), 709.

bioRxiv preprint doi: https://doi.org/10.64898/2026.04.10.717666; this version posted April 14, 2026. The copyright holder for this preprint (which was not certified by peer review) is the author/funder, who has granted bioRxiv a license to display the preprint in perpetuity. It is made available under aCC-BY 4.0 International license.

Supplementary Videos

Video S1: Animated description of Figure 3f
